# Brief Research Report on Adolescent Mental Well-Being and School Closures During the COVID-19 Pandemic in Indonesia

**DOI:** 10.3389/fpsyt.2020.598756

**Published:** 2020-11-17

**Authors:** Tjhin Wiguna, Gina Anindyajati, Fransiska Kaligis, Raden Irawati Ismail, Kusuma Minayati, Enjeline Hanafi, Belinda Julivia Murtani, Ngurah Agung Wigantara, Anggi Aviandri Putra, Kent Pradana

**Affiliations:** Faculty of Medicine Universitas Indonesia, Dr. Cipto Mangunkusumo General Hospital, Jakarta, Indonesia

**Keywords:** adolescent, mental health, mental wellbeing, Indonesia, COVID-19 pandemic, school closures, emotional problem, behavior problem

## Abstract

The COVID-19 pandemic does not affected only physical but also mental health and socioeconomic part. The social distancing, social quarantine, school from home, and work from becomes a new normal these days. Being adolescence, the above conditions may be challenging due to their developmental milestones. Therefore, this brief report aimed to preliminary identify proportion of adolescents' emotional and behavior problems and several factors related to it during COVID-19 pandemic in Indonesia. The findings might raise some understanding of youth mental well-being and programs that can be applied in schools and community in general to overcome the issues. The study was designed as cross sectional and used online survey that started on April 2020. During April 15–May 10, 2020, there were 113 adolescents participated on this survey. Strength and Difficulties Questionnaire (SDQ) 11–17 years old was used to assess adolescent emotional and behavior problems; and specific life experience questionnaire was designed to collect other independents variables (Cronbach's α = 0.75). All participants fulfilled the online informed consent before they started to complete the questionnaire. All data was analyzed by using SPSS version 20 for Mac. The average age of research subjects were 14.07 (2.18) years old; 98.2% was school from home. There was 14.2% of the total research subject at risk on total difficulties problems; 38.1% of adolescent was at risk on peer-relationship problems, 28.3% at risk on pro-social behavior problems, 15% at risk on conduct behavior and 10.6% at risk on emotional problems. The number of adolescent that perceived worse to significantly worse self-mental well-being prior COVID-19 increased during COVID-19 pandemic in Indonesia (*p* < 0.05). There was significantly association between having mental health information and conduct behavior (OR = 10.34, 95%CI = 1.27–78.86); Subjective anxiety due to COVID-19 pandemic and pro-social behavior problems (OR = 2.37, 95% CI = 1.00–5.63), parental support and total difficulties (OR = 0.09, 95% CI = 0.14–0.60) and pro-social behavior problems (OR = 0.09, 95% CI = 0.01–0.82); friends support during COVID-19 pandemic and conduct behavior (OR = 0.20, 95% CI = 0.04–1.00) and pro-social behavior problems (OR = 0.14, 95% CI = 0.02–0.75). To be concluded, during phase 1 and 2 COVID-19 pandemic and school closures in Indonesia, adolescents were at risk for having emotional and behavior problems. Therefore, maintain clear mental health information, keep them on connection with school by designing an optimal tele-education, tele-consultation, and virtual activity programs to accommodate adolescents' biopsychosocial needs in the near future.

## Introduction

The coronavirus disease (COVID-19) outbreak was declared a global pandemic in March 2020 (1), and it remains a major source of stress to all people. As of May 26, 2020, it is estimated that 5 million people in 190 countries have suffered from this illness, including children and adolescents, with over 300 000 deaths (2). Everybody and every country is facing hard times, fears, and various psychosocial problems. In response to this pandemic, there is a common trend regarding the social and physical distancing policies instituted, that is, the closure of schools, offices, and other public places to reduce viral transmission among people and students (3–6). In addition, several studies have revealed that school closures due to social and physical distancing also influence children and adolescents' mental well-being (7, 8). Based on the latest data (May 24, 2020), there are 22 271 COVID-19 cases in Indonesia, with ~40% in Jakarta, the capital city (9). However, the exact number of children and adolescents that have suffered from this illness in Indonesia is still unknown, and the definite number of school closures is unclear.

Similar to 188 countries around the world (5, 10), Indonesia has closed almost all schools, especially in contagious areas such as Jakarta, Central Java, West Java, and East Java. Children and adolescents have been advised to stay and study from home since the end of March 2020. Students have been asked to follow home-based distance-learning models; however, these kinds of models have not been standardized for kindergarten, elementary, middle, and high schools in Indonesia. Additionally, given the need for social distancing during the COVID-19 pandemic, people have been directed to stay and work from home; the Indonesian government has partially or totally closed down many offices, sports clubs, shopping malls, and other related businesses. Hence, social distancing measures have been associated with school isolation, social, and economic downturns (such as being expelled from work), unemployment, and increased child maltreatment cases due to parents working from home or even not working at all (6, 11–13). Moreover, information regarding the COVID-19 pandemic on social media is very chaotic, which in turn causes the public health crisis as it influences parents, children, and adolescents' perspectives on the pandemic situation, particularly the negative ones. Consequently, it leads to emotional contagion especially among adolescents because of the limited capability to assimilate and accommodate such exaggerated information (14, 15). Meanwhile, adolescent coping mechanisms are being developed and the influence of psychosocial turmoil; thus, they become more at risk of developing emotional and behavioral problems.

Adolescence is the period transition from childhood into adulthood. Based on the United Nation definition, adolescence is the period of between 10 and 19 years of age (16). It is a period to develop self-image, self-identity, self-certainty, self-esteem, and independence. Consequently, adolescence period incorporates biological and psychosocial factors. The brain hormonal changes in this period links to a gap or asynchrony between cognitive abilities, emotional regulation, and self-control capabilities. Furthermore, the need of fitting in and conforming to the standards of psychosocial roles of transition are very apparent during this period of development. Hence, adolescents experience inflated sense of self-esteem, self-importance, and uniqueness but at the same time they experience self-criticism, sadness, and anger (17). From the above development perspectives, adolescents may experience several challenging life situations such as, to achieve self-independence, academic competence, and future directions of professional existence. These conditions are associated to interpersonal conflicts that can increase the vulnerability to emotional problems (e.g., Depression, fear, worry, and anxiety). Moreover, it is also said to precipitate behavior problems such as, pro-social difficulties (e.g., Lack of emphaty and seldom share with others), conduct and disrupted acts, hyperactivity (e.g., Restless and difficult to stay focus), and peer-relationship problems (18). Based on the World Health Organization (WHO) data on 2016, the incidence rate of behavioral and emotional problems was higher in the age group >15 years and it was stated that 1 of 5 adolescents aged <16 years had those problems (19). Polanczyk et al. revealed that 11.8% of children and adolescents had mental health problems in Asian countries with low-income economies (20). On 2018, the Indonesian Ministry of Health reported that 9.8% of people age >15 years of age categorized as having mental emotional problems based on Self-Reporting Questionnaire-20 (21).

Thus, being a teenager is difficult, and it is assumed that the COVID-19 pandemic has made it even harder. For instance, to acquire the psychosocial strength to support their mental well-being, adolescents need to spend more time with their peers, engaging in outdoor activities and spending more time at school to accommodate their cognitive development and life skills (16, 17). Therefore, stay at home means they are missing sports activities, class activities, and many other important moments. Furthermore, schools are also established as supportive environments for youth; adolescents interact with their peers and teachers at school, sharing feelings, and thoughts (13, 22). Schools also play a role as a mental health delivery agent. Consequently, being at home forces them to adapt and struggle, and somehow becomes a psychosocial stressor. Examining youth mental health issues has become more essential; especially because students cannot access their school due to the pandemic, they might lose enthusiasm in every aspect of their lives. A study by Zanonia Chu, as cited by Lee (10), a psychologist in Hong Kong, found that many adolescents showed emotional, pro-social problems, and conduct behavior such as, isolated themselves in their cubicle for weeks, refusing to clean, eat, or leave their room after the schools were closed down during the COVID-19 outbreak in Hong Kong (10). Therefore, it is essential to emphasize adolescent mental well-being during this COVID-19 pandemic, especially related to school closures.

This brief report aimed to preliminary identify the proportion of adolescent at risk of behavioral and emotional problems, and to elaborate the association between several factors related to it during the phase one and two of COVID-19 pandemic in Indonesia. Furthermore, Indonesia has hardly been affected by COVID-19; especially in the last ten decades, Indonesia never experiences infectious diseases outbreak like COVID-19 pandemic. Therefore, these preliminary findings might provide a better understanding of youth mental health during the COVID-19 pandemic, especially related to programs that can support youth mental well-being and can be employed to increase adolescent coping strategies and learning capabilities. Moreover, these brief findings can be referred to other countries in terms of organizing adolescent mental well-being during the first and second phases of a pandemic.

## Methods

This study is part of an ongoing community survey on mental health during the COVID-19 pandemic in Indonesia; it is a cross-sectional study using an online survey approach. The inclusion criteria were family with parents with at least a junior high school background and children aged 11–17 years. All families and adolescents gave their consent to participate in this study by signing an online informed consent form. The Ethics Committee of the Faculty of Medicine of Universitas Indonesia approved the study protocol in April 2020. During the period of April 15 to May 10, 2020 there were 213 questionnaires that were filled out by the adolescents, however only 113 questionnaires that were fully completed and thus were included in the study analysis. The study protocol was registered to ClinicalTrials.gov with ID NCT04343664.

### Instrument

In addition to the demographic data of both parents and adolescents, the study developed a specific questionnaire related to life experiences during the COVID-19 pandemic that was completed by adolescents themselves, which comprised questions such as “How do you perceive your mental well-being before and during the COVID-19 pandemic?”; “Are you schooling from home?” and “if ‘Yes,' for how many days from now?”; “Do you get any support (from family or friends) during this period of the COVID-19 pandemic?”; “Do you get any health or mental health information during the COVID-19 pandemic?”; and “Is there any lifestyle change due to the COVID-19 pandemic?” Several questions were answered with “Yes” or “No,” and several others were on their subjective experiences. Further, the participants were asked to rate their subjective anxiety due to the COVID-19 pandemic; the scoring was determined using an analog scale ranging from 0 to 10, with a higher score indicating that they were more worried about it. The questionnaire had a good reliability for community research with Cronbach's α = 0.75.

The study used the Strength and Difficulties Questionnaire (SDQ) adolescent version (self-rating) to assess emotional and behavioral problems that can be used in the context of social sphere. SDQ has been translated into Indonesian language and back translated as well into the original language (English) by Wiguna and Hestyanti; it is available at www.sdqinfo.com. The SDQ consists of 25 items on a Likert scale (0 = not true;1 = somewhat true;2 = certainly true), and comprises two major domains: (1) difficulties domains (consisted of 20 items) that include emotional problems, hyperactivity behavior, peer-relationship problems, and conduct behavior, and (2) pro-social behavior (five items) as the strength domain. The total score for the difficulties domains ranged from 0 to 40, it can also categorized as total score 0–14 indicates normal, a total score 16–19 indicates borderline and 20–40 indicates an abnormal score (23). Due to the purpose of the study, the cut-off of ≥15 was set to include all those who scored borderline and above (at risk group) and a total score <15 wa categorized as normal group. Community-based studies recommended the 90th percentile as the threshold for various child psychiatric screening tools (24). Using a cut-off of 14 equates to the 90th percentile, it yields excellent specificity (91%) and reasonable sensitivity (70%) (23). However, this study also looked at each difficulties domain: the higher the score of each difficulty domain, the higher the risk of getting those problems. Each difficulty domain was categorized into normal group and at-risk group respectively; (1) emotional problems (a total score < 6 = normal group and ≥6 = at risk group); (2) hyperactivity behavior (a total score < 6 = normal group and ≥ 6 = at risk group); (3) peer-relationship problems (a total score < 4 = normal group and ≥ 4 = at risk group); (4) conduct behavior (a total score < 4 = normal group and ≥ 4 = at risk group). The total score for the strength domain (prosocial behavior) is 0–10: the higher the score of the prosocial behavior, the lower the risk for pro-social behavior problems (23). A total score of prosocial behavior >5 was categorized as normal group, and ≤5 was at risk group.

### Data Analysis

Data analysis was performed using SPSS version 21 for Mac. The data comprised numerical and categorical variables; therefore, to identify the association between each dependent and independent variable, all numerical data were converted into bivariate categorical data. Two independent variables (number of days schooling from home and subjective anxiety due to COVID-19) were categorized as below and above the mean. The Chi-Square test, Fisher's exact test, and Spearman's rank correlation test were applied to elaborate the association, and the *p*-value was set to < 0.05.

## Results

During April 15 and May 10, 2020, 113 adolescents aged 11–17 years had completed the entire questionnaire. The mean age (SD) was 14.07 (2.18) years; most had a junior or senior high school background, and the number of boys was comparable to that of girls (53.1 vs. 46.9%). Most of them (98%) had been schooling from home, and the duration of schooling ranged from 6 to 60 days, with an average of 26.58 days. Originally, the research subjects were from middle-high income families (70.6%). The study found that 66.4% of the total research subjects had health or mental health information during the COVID-19 pandemic from social media, friends, or newspapers. In addition, the number of research subjects that revealed lifestyle changes during the COVID-19 pandemic was around 50%. In all, 10.6% of research subjects were at risk for emotional problems, 15.0% for conduct behavior, 38.1% for peer-relationship problems, 8% for hyperactivity behavior, and 28.3% for pro-social behavior problems (Tables 1–3).

**Table 1:** Characteristic of research subjects.

**Characteristics**	**Mean (SD)**	**Range**	***n* (%)**	**95% Confidence Interval**
Age (year)	14.07 (2.18)	11–17		13.64–14.50
≤ 15 years old			76 (67.3)	58.4–77.0
>15 years old			37 (32.7)	23.0–41.6
Gender				
Boy			60 (53.1)	43.4–62.8
Girl			53 (46.9)	37.2–56.6
Educational background				
Elementary school			25 (22.1)	15.0–30.1
Junior high school			48 (42.5)	33.6–52.2
Senior high school and above			40 (35.4)	25.7–44.2
School from home				
Yes			111 (98.2)	95.6–100.0
No			2 (1.8)	0–4.4
Number of days schooling from home	26.58 (6.96)	6–60		24.80–28.32
≤ Mean			61 (53.9)	
>Mean			52 (46.1)	
Subjective anxiety due to Covid-19 pandemic	3,57 (2.27)	1–10		3.13–4.04
≤ Mean			94 (83.2)	
> Mean			19 (16.8)	
Emotional problems	1.69 (1.80)	0–8		1.36–2.04
Normal			101 (89.4)	83.2–94.7
At risk			12 (10.6)	5.3–16.8
Conduct behavior	1.88 (1.43)	0–8		1.63–2.19
Normal			96 (85.0)	77.9–91.2
At risk			17 (15.0)	8.8–22.1
Hyperactivity	2.36 (1.85)	0–7		2.03–2.72
Normal			105 (92.9)	87.6–97.3
At risk			8 (7.1)	2.7–12.4
Peer relationship problems	2.00 (1.54)	0–6		1.71–2.31
Normal			70 (61.9)	77.0–91.2
At risk			43 (38.1)	8.8–23.0
Pro-social behavior problems	7.70 (1.86)	4–10		7.36–8.06
Normal			81 (71.7)	86.7–96.5
At risk			32 (28.3)	2.5–13.3
Total difficulties problem	8.25 (4.94)	2–25		7.35–9.3
Normal			97 (85.8)	79.6–92
At risk			16 (14.2)	8.0–20.4

**Table 2:** Characteristic of life experiences among research subjects (*n* = 113).

**Characteristics**	***n* (%)**	**95% confidence interval**
**Life style changes during Covid-19 pandemic**		
Yes	57 (50.4)	41.6–59.3
No	56 (49.6)	40.7–58.4
**Support from family during the Covid-19 pandemic**		
Satisfied	109 (96.5)	92.9–99.1
Dissatisfied	4 (3.5)	0.9–7.1
**Support from friends during the Covid-19 pandemic**		
Satisfied	107 (94.6)	90.3–98.2
Dissatisfied	6 (5.4)	1.8–9.7
**Having health or mental health information during Covid-19 pandemic**		
Yes	75 (66.4)	57.5–75.2
No	38 (33.6)	24.8–42.5

**Table 3:** Characteristics of parental background.

**Characteristics**	***n* (%)**	**95% confidence interval**
**Socio-economic background (*****n*** **=** **112)**		
Middle-low income	28 (25.0)	17.9–33.0
Middle-high income	84 (75.0)	67.0–82.1
**Parents work from home (*****n*** **=** **112)**		
Yes	74 (66.1)	57.1–75.0
No	38 (33.9)	25.0–42.9
**Number of hours for parents work from home (*****n*** **=** **113)**		
<6 h/day	89 (78.8)	70.8–85.8
≥7 h/day	24 (21.2)	14.2–29.2
**Social distancing (*****n*** **=** **112)**		
Yes	109 (97.3)	93.8–100.0
No	3 (2.7)	0–6.3

Nevertheless, there were a significant association between subjective experienced toward self-mental well-being before- and after COVID-19 pandemic (p < 0.05). Consequently, the subjective experience on their own mental well-being during the COVID-19 pandemic were significantly correlated with emotional problems (r = 0.31, 95% CI = 0.13–0.49), hyperactive behavior (r = 0.33, 95% CI = 0.14–0.48), peer-relationship problems (r = 0.32, 95% CI = 0.15–0.48), and pro-social behavior problems (r = −0.42, 95% CI = −0.57–−0.26); they were also correlated with conduct behavior, but it was not statistically significant (r = 0.16, 95% CI = −0.42–0.33).

This study showed that gender, life style changes during the COVID-19 pandemic, health or mental health information during the pandemic, number of days schooling from home, subjective perception toward anxiety due to the COVID-19 pandemic, and parental and friend support during the pandemic were associated with emotional problems, conduct behavior, hyperactive behavior, peer-relationship problems, and pro-social behavior problems. However, only a few associations were statistically significant, such as having health or mental health information during the COVID-19 pandemic and conduct behavior (OR = 10.34, 95% CI = 1.27–78.86); subjective perception toward anxiety due to COVID-19 and pro-social behavior problems (OR = 2.37, 95% CI = 1.00–5.63); parental support during the pandemic and total difficulties problems (OR = 0.09, 95% CI = 0.14–0.60) and pro-social problems (OR = 0.09, 95% CI = 0.01–0.82); friend support during the pandemic and conduct behavior (OR = 0.20, 95% CI = 0.04–1.01); friend support during the pandemic and pro-social behavior problems (OR = 0.14, 95% CI = 0.02–0.75) (Table 4).

**Table 4:** The association between independent variables and adolescents emotional and behavior problems.

**Characteristics**	**Total difficulties problems**		**OR (95% CI)**	**Emotional problems**		**OR (95% CI)**	**Conduct problems**		**OR (95% CI)**	**Hyperactivity**		**OR (95% CI)**	**Peer-relationship problems**		**OR (95% CI)**	**Pro-social behavior problems**		**OR (95% CI)**
	**At risk**	**Normal**		**At risk**	**Normal**		**At risk**	**Normal**		**At risk**	**Normal**		**At risk**	**Normal**		**At risk**	**Normal**	
**Gender:**																		
Boys (Ref)	10	50	1.57	4	56	0.40	10	50	1.31	4	56	0.87	26	34	1.62	17	43	1.00
Girls	6	47	(0.53–4.65)	8	45	(0.11–1.42)	7	46	(0.46–3.73)	4	49	(0.21–3.69)	17	36	(0.75–3.45)	15	38	(0.44–2.27)
**Life style changes due to Covid−19 pandemic:**																		
Yes (Ref)	7	50	1.37	4	51	1.89	8	49	1.17	5	52	0.60	24	33	0.71	15	42	1.22
No	9	47	(0.47–3.97)	7	46	(0.52–6.87)	9	47	(0.41–3.29)	3	53	(0.13–2.59)	19	37	(0.33–1.51)	17	39	(0.54–2.77)
**Having health or mental health information during Covid−19 pandemic:**																		
Yes (Ref)	12	63	1.62	10	65	2.77)	16	59	10.34^*^	6	69	1.56	30	45	1.28	24	51	1.76
No	4	34	(0.48–5.41)	2	36	(0.57–13.33	1	37	(1.27–78.86)	2	36	(0.12–3.33)	13	25	(0.57–2.89)	8	30	(0.70–4.42)
**Number of days that was school from home:**																		
≤ Mean (Ref)	9	46	1.53	4	51	0.51	10	45	2.13	2	53	0.36	27	28	2.69^*^	17	38	1.71
>Mean	6	47	(0.50–4.65)	7	46	(0.14–1.87)	5	48	(0.68–6.72)	5	48	(0.07–1.95)	14	39	(1.20–6.02)	11	42	(0.71–4.10)
**Subjective anxiety due to Covid−19 pandemic:**																		
≤ Mean (Ref)	10	51	0.67	5	56	0.57	9	52	0.95	5	56	1.45	23	38	0.97	22	39	2.37^*^
> Mean	6	46	(0.22–1.97)	7	45	(0.17–1.93)	8	44	(0.34–2.68)	3	49	(0.33–6.42)	20	32	(0.45–2.07)	10	42	(1.00–5.63)
**Parental support during Covid−19 pandemic:**																		
Satisfied (Ref)	13	95	0.09^**^	11	97	0.45	15	93	0.24	7	101	0.28	39	69	0.14	28	80	0.09^**^
Dissatisfied	3	2	(0.14–0.60)	1	4	(0.05– 4.43)	2	3	(0.04–1.57)	1	4	(0.03–2.82)	4	1	(0.01–1.31)	4	1	(0.01–0.82)

* *Chi-square test with p < 0.05*.

** *Fisher's exact test with p < 0.05*.

## Discussion

This brief research report was presented data during the first and second phases of the pandemic, which was defined as the period between the Indonesian government's enforced social distancing, school closures, and personal hygiene measures such as hand washing and the use of face masks, in order to mitigate the spread of the infection, and the period of highest incidence of new cases and mortality rate peaks (25, 26). A study conducted in China in 2020 mentioned that 82% of their participants used social media as the main source of information during the COVID-19 pandemic, and it was highly associated with anxiety and depression (20). Therefore, various health and general information related to COVID-19 might not be originally derived from definite sources; such information spread through social media or chat rooms and included false information. In addition, many people (including adolescents) used social media to express their frustration and feelings, such as fear, worry, nervousness, and anxiety (27). This put the local community under immense pressure since it might trigger negative emotional states. Negative emotional states can be transferred to adolescents through emotional contagion processes and lead them to experience similar negative emotions (14).

In this present report, most of the participants accessed social media to obtain health or mental health information. Hence, it revealed that adolescents perceived that COVID-19 pandemic exposure highly affected their mental well-being such as became slightly worse to significantly worse than before the COVID-19 pandemic. It was also positively associated with their mental well-being, such as emotional and peer-relationship problems, and negatively correlated to pro-social behavior skills. In addition, this brief report found that unnecessary health or mental health information during the 1st and 2nd phase of COVID-19 pandemic might become a risk for adolescents to develop emotional and behavior problems.

The findings of this brief study report also showed that the proportion of adolescents at risk for peer-relationship problems, pro-social behavior problems and conduct behavior was quite high. However, the proportion of emotional problems among the study participants was not as high as the average proportion of emotional problems in community samples outside the COVID-19 pandemic (28). Ortuno-Sierra et al., reported the patterns of behavioral and emotional difficulties from 1,474 adolescents at North Spain by using SDQ, and found that 12.16% of the total subject experienced emotional problems, 10.36% had conduct problems, 14.36% experienced hyperactivity behavior, 9.26% with peer-relationship problems, and 20.34% had difficulties in pro-social behavior (18). Another study from Malaysia on 2019 reported that 12.3% of the total adolescent at risk for emotional problems and conduct behavior, 12.9% at risk for hyperactivity behavior, 18.3% at risk for peer-relationship problems and 23.4% at risk for pro-social behavior (29). Therefore, the results of those two studies that originally from two different parts of the world showed a quite various proportion on behavioral and emotional problems among adolescents prior the COVID-19 pandemic.

This preliminary result might be related to the fact that most participants in this study felt satisfied with the parental and friend support during the COVID-19 pandemic. Hence, it might be a protective factor against being at risk for emotional problems. On the other hand, this report revealed that feeling dissatisfied with parental and friend support during the COVID-19 pandemic increased the risk for total difficulties problems, conduct behavior, peer-relationship problems, and pro-social behavior problems. Therefore, even though most participants received parental and friend support, lifestyle changes due to the COVID-19 pandemic are still difficult for them to cope with.

The participants and their families were forced to stay at home during COVID-19 as suggested by the Indonesian government policy and limit their leisure-time activities because schools, public places and several working places were locked down. In many countries affected by COVID-19, adolescents have been prohibited from going to playgrounds, engaging in direct group activities, and mingling in public places, and sport yards/clubs/malls have been closed (30). People (including adolescents in this study) were strongly asked to limit their social interactions, except with the closest family members; they were prohibited from meeting their peers. Adolescence is a period of rational thinking development due to the immaturity of the prefrontal cortex (31). Thus, social-emotional networks are much more dominant in their regular acts; therefore, various COVID-19 information (both factual and false), school closures, and social distancing negatively affect adolescents because being rational and peer contact are important for maintaining mental well-being (32, 33). Another study showed that school closures might not lead to a reduction in infections and the prevention of deaths (5). Consequently, potential harmful consequences such as deficits in schooling time, delay of exams, limited socialization with peers, restrictions regarding daily activities outside the home, and emotional and behavioral problems need to be taken into account when assessing the advantages and disadvantages of this particular measure to adolescent mental well-being (25).

At the parental level, the COVID-19 pandemic has led to a restructuring of daily life. All family members have to cope with the stress of social distancing, schooling from home, working from home, and staying at home. Most parents felt pressure working from home, keeping jobs and businesses running as well as assisting children and adolescents to study from home at the same time. Thus, family support may be limited and it increased risk of child and adolescent maltreatment such as abuse and neglect as well (34). Another study explained that parents had a high burden because they needed to explain the COVID-19 pandemic to children and adolescents; however, they should handle their own fear and anxiety accompanying these uncertain times (25).

This was the first report to focus on adolescent mental health during the first and second phases of the COVID-19 pandemic in Indonesia. The results highlight several challenges regarding child and adolescent mental well-being during this period in Indonesia, such as maintaining support for adolescents from families and friends, how to regulate their subjective feeling toward the COVID-19 pandemic, and delivering appropriate health and mental health information related with COVID-19 pandemic. Therefore, it is necessary to provide clear and correct information (health, mental health and psychoeducational facts) to family members and adolescents themselves. Moreover, a better understanding of COVID-19 is important to prevent uncomfortable or negative feelings and help comply with government rules and policies. Information can be delivered through psychoeducation and consists of parenting skills, such as how to communicate with adolescents about COVID-19; negative feelings and thoughts that trouble them; associated risks regarding hand hygiene, self-caring, and using face masks/shields; and changes in daily lifestyles. It should also cover how to manage schooling-from-home activities and schedules, how to handle adolescent stress and anxiety, and how to organize their daily activities outside school. Additionally, information about early detection of adolescent mental health problems should be provided so that parents could notice it when necessary; using self-rating questionnaires such as SDQ or other self-rating instruments can be useful. The referral pathway also needs to be generated for families and communities in general, including therapeutic approaches such as psycho-pharmacotherapy, crisis intervention, or any psychotherapy approach when needed. Using social media or web-based platforms is being necessary because of social distancing and other restrictions during phase one and two of the pandemic. Child and adolescent psychiatrists play an important role in facing this pandemic by supporting the continuity of care; leading parents, teachers, children, adolescents, and other health and mental health professionals on how to manage mental health issues; and elucidating the biopsychosocial impact of the pandemic on the well-being of children and adolescents.

To keep connecting with peers can be a supportive condition for adolescents during the COVID-19 pandemic (10). Therefore, schooling from home can be facilitated by a sophisticated design of tele-education/e-learning policies. This can be a good approach during this pandemic period. Nowadays, adolescents are enthusiastic about modern technology, and it is assumed to constitute a substantial part of their social and educational lives (35). Furthermore, by implementing tele-education, adolescents can maintain the feeling of being at school and connect with their peers even when they are all at home. However, modern educational technology requires good teamwork between school administrators, technology coordinators, teachers, students, parents, and the community in general (35).

On the other hand, maintaining supports for adolescents and families can be delivered by telepsychiatry services. This consultation care model can be run in the primary care setting, where the primary care physician provides a mental health service and counseling or supportive psychotherapy as needed. The child and adolescent psychiatrist can also work with local medical doctors and local medical team members such as nurses, midwives, and psychologists to provide recommendations and training for local medical doctors or medical team members in specific skills and knowledge (36–38). Several studies have mentioned that child and adolescent telepsychiatry, such as online-delivered psychotherapeutic care for children and adolescents, is feasible and effective (39–43); therefore, it can be considered as the primary source of standard care in times of restricted physical contact.

To conclude, this brief research report presented several important data on adolescent well-being during the COVID-19 pandemic in Indonesia especially during the first and second phase of pandemic. In addition, tele-education can be one or several other choices that can maintain adolescent daily activities and keep them connect to their peers. In addition, supportive psychotherapy and psychoeducation for parents, adolescents and other family members can be delivered through telepsychiatry services since the COVID-19 pandemic still goes on and nobody knows when it would end. However, it needs to be adjusted in local low resources places. The study revealed several limitations: first, this brief report only covered adolescents that could access Internet. Second, it did not include other factors that might affect the outcome, such as screen time, recent social activities, chronic illness, number of hours of study at home per day, adolescent stress and coping strategies, and parental stress that might have correlation with emotional and behavior problems among adolescents during the first and second phases of the COVID-19 pandemic. Therefore, further publication with more participants and more related factors should be done to gain a better understanding of adolescent mental health during this hard time.

## Data Availability Statement

The raw data supporting the conclusions of this article will be made available by the authors, without undue reservation.

## Ethics Statement

The studies involving human participants were reviewed and approved by The Ethics Committee of the Faculty of Medicine of Universitas Indonesia. Written informed consent to participate in this study was provided by the participants' legal guardian/next of kin.

## Author Contributions

TW contributing author, designing, analyzing, writing the results, and discussion. GA, BM, and KM designing and results analyzing. FK and RI writing discussion. EH designing and contributing the review process. NW designing and contributing in review the writing. AP designing and contributing the analyzing. KP editing the document and contributing in the table analyzing. All author contributed equally for this paper.

## Conflict of Interest

The authors declare that the research was conducted in the absence of any commercial or financial relationships that could be construed as a potential conflict of interest.
